# Assessment of precision in growth inhibition assay (GIA) using human anti-PfRH5 antibodies

**DOI:** 10.1186/s12936-023-04591-6

**Published:** 2023-05-19

**Authors:** Kazutoyo Miura, Ababacar Diouf, Michael P. Fay, Jordan R. Barrett, Ruth O. Payne, Ally I. Olotu, Angela M. Minassian, Sarah E. Silk, Simon J. Draper, Carole A. Long

**Affiliations:** 1 Laboratory of Malaria and Vector Research, National Institute of Allergy and Infectious Diseases, National Institutes of Health, 12735 Twinbrook Parkway, Rockville, MD 20852 USA; 2 Biostatistics Research Branch, National Institute of Allergy and Infectious Diseases, National Institutes of Health, Rockville, MD 20852 USA; 3 Department of Biochemistry, University of Oxford, Dorothy Crowfoot Hodgkin Building, Oxford, OX1 3QU UK; 4 Interventions and Clinical Trials Department, Ifakara Health Institute, P.O. Box 74, Bagamoyo, Tanzania

**Keywords:** Growth inhibition assay, RH5, *Plasmodium falciparum*, Vaccine, Blood-stage, Precision

## Abstract

**Background:**

For blood-stage malaria vaccine development, the in vitro growth inhibition assay (GIA) has been widely used to evaluate functionality of vaccine-induced antibodies (Ab), and *Plasmodium falciparum* reticulocyte-binding protein homolog 5 (RH5) is a leading blood-stage antigen. However, precision, also called “error of assay (EoA)”, in GIA readouts and the source of EoA has not been evaluated systematically.

**Methods:**

In the Main GIA experiment, 4 different cultures of *P. falciparum* 3D7 parasites were prepared with red blood cells (RBC) collected from 4 different donors. For each culture, 7 different anti-RH5 Ab (either monoclonal or polyclonal Ab) were tested by GIA at two concentrations on three different days (168 data points). To evaluate sources of EoA in % inhibition in GIA (%GIA), a linear model fit was conducted including donor (source of RBC) and day of GIA as independent variables. In addition, 180 human anti-RH5 polyclonal Ab were tested in a Clinical GIA experiment, where each Ab was tested at multiple concentrations in at least 3 independent GIAs using different RBCs (5,093 data points). The standard deviation (sd) in %GIA and in GIA_50_ (Ab concentration that gave 50%GIA) readouts, and impact of repeat assays on 95% confidence interval (95%CI) of these readouts was estimated.

**Results:**

The Main GIA experiment revealed that the RBC donor effect was much larger than the day effect, and an obvious donor effect was also observed in the Clinical GIA experiment. Both %GIA and log-transformed GIA_50_ data reasonably fit a constant sd model, and sd of %GIA and log-transformed GIA_50_ measurements were calculated as 7.54 and 0.206, respectively. Taking the average of three repeat assays (using three different RBCs) reduces the 95%CI width in %GIA or in GIA_50_ measurements by ~ half compared to a single assay.

**Conclusions:**

The RBC donor effect (donor-to-donor variance on the same day) in GIA was much bigger than the day effect (day-to-day variance using the same donor’s RBC) at least for the RH5 Ab evaluated in this study; thus, future GIA studies should consider the donor effect. In addition, the 95%CI for %GIA and GIA_50_ shown here help when comparing GIA results from different samples/groups/studies; therefore, this study supports future malaria blood-stage vaccine development.

**Supplementary Information:**

The online version contains supplementary material available at 10.1186/s12936-023-04591-6.

## Background

Reticulocyte-binding protein homolog 5 (RH5) is expressed on merozoites of *Plasmodium falciparum,* which is the most lethal *Plasmodium* species that causes malaria in humans, and binds to basigin on the surface of erythrocytes [1]. The RH5 forms a complex with the RH5-interacting protein (Ripr) and the Cysteine-rich protective antigen (CyRPA) [2]. Formation of this complex [3] and binding between RH5 and basigin [1] are essential steps during parasite invasion. Thus, RH5, CyRPA and Ripr are current leading blood-stage vaccine candidates [4]. A recent study has shown the three antigens form a pentameric complex with two additional antigens, *P. falciparum* Plasmodium thrombospondin-related apical merozoite protein (PfPTRAMP) and *P. falciparum* cysteine-rich small secreted protein (PfCSS) [5].

In a *P. falciparum* challenge model using *Aotus* monkeys, both RH5 vaccination [6] and anti-RH5 monoclonal antibody (mAb) inoculation [7] induced protection. To define the mechanism of protection, the latter study utilized a mutated mAb, c2AC7, which did not engage complement or FcR-dependent effector mechanisms, and showed that the mutated mAb could induce protection [7]. In the two *Aotus* challenge studies, significant positive correlations were observed between functional activity of antibodies measured by in vitro growth inhibition assay (GIA) and in vivo protective effects [6, 7]. In addition to the *Aotus* data, a positive correlation has been also seen in humans [8]. In a Phase I/IIa trial, malaria-naïve UK adults were vaccinated with recombinant RH5.1 protein formulated with AS01_B_ adjuvant, then controlled human malaria infection (CHMI) was conducted using blood-stage parasites; in vivo efficacy was assessed as a reduction in *P. falciparum* blood-stage multiplication rates (in vivo growth inhibition). There was a significant correlation between in vitro GIA activity before the challenge and in vivo growth inhibition (spearman coefficient = 0.60, p = 0.0001) [8].

GIA has been widely used to evaluate vaccine-induced functional immunity against a variety of blood-stage candidates, such as apical membrane antigen 1 (AMA1), merozoite surface protein 1 (MSP1), and erythrocyte binding antigen 175 (EBA-175), in animals and humans [9]. Furthermore, immunization of *Aotus* with AMA1 [10] or MSP1 [11] followed by challenge demonstrated significant correlations between in vivo protection against blood-stage *P. falciparum* and in vitro GIA. The significant correlations seen in multiple non-human primate and human studies strongly support the idea that GIA is a valuable tool to evaluate functionality of vaccine-induced antibodies in preclinical and early clinical development of blood-stage vaccines, especially for RH5-based vaccines. Using the GIA, vaccine developers may compare efficacy between current and newly engineered RH5 recombinant proteins, between current and new vaccine formulations, and so on. However, for such quantitative comparisons, instead of qualitative judgement (e.g., whether or not a new vaccine candidate can induce GIA-positive antibodies), assessment of “error of assay (EoA)” in GIA readout is essential. EoA in this manuscript denotes variability in GIA readouts when the same sample is repeatedly tested on different days, in different plates, by different operators, and/or using different red blood cell (RBC), and it refers to both the variance of the assay as well as functions of that variance (e.g., standard deviation (sd) or 95% confidence interval (95%CI) widths for averages of replicates). EoA does not indicate a technical error (e.g., a wrong sample was tested at a wrong concentration).

EoA in % inhibition in GIA (%GIA) readout was previously evaluated in order to develop a general statistical method of analysis [12]. However, in that study, only a single rabbit anti-AMA1 antibody was tested at serial dilutions in 4 independent assays. The limited data did not allow a more complete analysis of statistical dependence, and %GIA data from different concentrations in a single assay were treated as independent readouts. In this study, 7 different samples were tested at 2 concentrations using 4 different batches of RBC on three different days (168 data points) to evaluate effects of RBC donor and day of assay on EoA more precisely. In addition, with a larger data set (5,093 data points from 180 samples collected from three human clinical trials), the sd and 95%CI in %GIA and GIA_50_ (antibody concentration that gives 50%GIA) measurements in single and multiple assays were calculated.

## Methods

### Reagents

Two rabbit Protein-G-purified anti-RH5 polyclonal antibodies (pAb) were obtained from a previous study [13], and the details of three human anti-RH5 monoclonal antibodies (mAb), R5.004, R5.008 and R5.016, were described previously [14]. Human serum (Blood type O, Rh +) and RBC (Blood type O, Rh +) collected from malaria naïve US adults were purchased from Interstate Blood Bank (Memphis, TN, USA) for malaria culture and GIA. The incomplete culture medium (RPIM1640 with L-Glutamine + 25 mM Hepes + 50 mg/mL Hypoxanthine) was obtained from KD Medical (Columbia, MD, USA). Sodium bicarbonate, gentamicin, 1 × phosphate buffered saline (PBS) and 1 M Tris–HCl (pH8.0) were obtained from Thermo Fisher Scientific. Diaphorase from *Clostridium klyiveri* (*Ck*D), Triton X-100, nitro blue tetrazolium (NBT) tablets, acetylpyridine adenine dinucleotide (APAD), and L ( +)-sodium lactic acid salt were purchased from Sigma-Aldrich.

### Human trials with RH5 vaccines

Human anti-RH5 sera were collected from three clinical trials (VAC057, VAC063 and VAC070), and total IgG was purified using a Protein G column (Cytiva; Marlborough, MA, USA) for each serum sample following manufacturer’s instructions. In all three trials, written informed consent was obtained from study participants or the parents or guardians of children aged < 18 years.

The details of VAC057 (ClinicalTrials.gov Identifier NCT02181088) [15] and VAC063 (NCT02927145) [8] have been reported previously. In brief, healthy UK adults were immunized with full-length RH5 using a chimpanzee adenovirus serotype 63 and modified vaccinia virus Ankara (ChAd63-MVA) vaccination platform in the VAC057 trial, or with a full-length recombinant RH5 protein (called “RH5.1”) formulated in GlaxoSmithKline’s adjuvant system AS01_B_ in the VAC063 trial.

VAC070 trial (ClinicalTrials.gov Identifier NCT03435874; Pan African Clinical Trials Registry, PACTR20171000272229; and ISRCTN47448832) was a dose-escalation, age de-escalation randomized double-blind controlled Phase 1b study conducted in Tanzania. In the trial, adults (18–35 years), young children (1–6 years) and infants (6–11 months) were immunized with the same ChAd63-MVA vaccines, as in the VAC057 trial. The adults received 5 × 10^10^ viral particles (vp) of ChAd63 on day 0 and 2 × 10^8^ plaque-forming units (pfu) of MVA on day 56. The young children and infants received ChAd63 followed by MVA on the same schedule with the same doses or a reduced dose combination (1 × 10^10^ vp of ChAd63 on day 0 followed by 1 × 10^8^ pfu of MVA on day 56). As a comparator, groups of participants received Rabies vaccine on days 0 and 56. The details of the trial are described elsewhere [16]. At the time of this GIA study, the sample identity was kept blinded to the analysts.

### Malaria culture and GIA

Malaria culture and GIAs were performed with the 3D7 clone of *P. falciparum* as described previously [17]. *Plasmodium falciparum* 3D7 parasites were maintained using complete culture medium (the incomplete culture medium plus 2.5 g/L of sodium bicarbonate, 10 mg/L of gentamicin and 10% pooled human serum) in an atmosphere of 5% O_2_, 5% CO_2_ and 90% N_2_ at 37 °C with periodic synchronizations either by percoll or sorbitol. The human sera were pools from 5 to 15 units of serum, and 4 different human serum pools were utilized for this study. However, the same serum pool was used for malaria culture and GIAs to test the same IgG samples in multiple assays. Before performing GIAs, the parasites were cultured for a minimum of 4 days with specified donor’s RBCs, then the day before assay, additional sorbitol synchronization was performed. On the day of GIA, the trophozoite-rich *P. falciparum* culture was diluted to ~ 0.3% parasitaemia, and mixed with a test pAb/mAb at an indicated concentration in a 96-well plate (Sterile 96-well flat bottom half well tissue culture plates, Corning, catalog number 3696). Each well contained 40 μL of complete culture medium and a test antibody (except for the control wells described next) with 1% haematocrit at ~ 0.3% parasitaemia. To determine % inhibition in GIA (%GIA), two controls (in triplicate wells) were included in each GIA plate; infected RBC alone without any test Ab (iRBC) and uninfected RBC alone (uRBC). The plate was covered by the lid, then incubated at the same atmosphere and temperature for ~ 40 h. After the incubation, the plate was washed with 120 μL/well of cold 1 × PBS three times, then 120 μL/well of parasite-specific lactate dehydrogenase (LDH) assay solution (0.1 M Tris–HCl with 25 mL/L of Triton X-100, 50 mg/L of APAD, 1 unit/L of *Ck*D, 56 g/L of L ( +)-sodium lactic acid, and 200 mg/L of NBT) were added. The plate was transferred to a VersaMax microplate reader (Molecular Devices Co., San Jose, CA, USA) and optical density at 650 (OD_650_) for each well was read for every 2 min. When iRBC alone control wells reached to OD_650_ of 0.4–0.5 (usually 8–12 min), the assay was completed.

### Main GIA experiment

A single *P. falciparum* 3D7 culture was split into four different cultures, and each culture was maintained using one of 4 batches of RBC collected from 4 different donors (Donor A, B, C and D). On day 12, using each culture, 2 human pAb (from VAC063 study), 2 rabbit pAb, and 3 human mAb were tested in triplicate wells by GIA at two concentrations (4 donors × 7 samples × 2 concentrations = 56 data points). A single GIA plate was used for each culture (7 test samples at 2 concentrations, plus two controls). The repeat assays were performed on days 14 and 22 (a total of 168 data points). The original %GIA values for each test sample in each assay are shown in Additional file 1: Table S1 (Study name is “Main” in Additional file 1: Table S1).

## Clinical GIA experiment

For VAC063 trial, the purified IgGs were tested at 10 mg/mL in triplicate wells first, then samples that showed more than ~ 40%GIA (n = 86 IgGs) were selected for titration GIA, where each sample was tested at twofold serial dilutions (from 10 to 0.039 mg/mL) in duplicate wells. The titration GIAs were performed three times, and a different batch of RBC was used to culture and perform GIA for each assay (i.e., each IgG sample was tested with 4 different batches of RBCs at 10 mg/mL, and with 3 different RBCs at lower concentrations). If %GIA of a test IgG at 10 mg/mL was higher than 50 in all three titration GIAs, GIA_50_ (antibody concentration that gave 50%GIA) for each assay was calculated (n = 58 IgGs). The assays were conducted using RBCs from 8 donors (donor E to J, W and X) on 28 different days (Study name of “VAC063” in Additional file 1: Table S1). A total of 2,404%GIA values and 174 GIA_50_ values were calculated from this trial.

For VAC070 trial, each purified IgG was tested at the physiological concentration of total IgG in the original serum first (in duplicate wells), then 3 independent titration GIAs (from physiological concentrations to up to 1:512 dilution; duplicate wells or singlicate well, depending on the available volume of each purified IgG) were performed for the IgGs with more than ~ 40%GIA in the first assay (n = 73 IgGs). The assays were conducted using RBCs from 8 donors (donor K to P, Y and Z) on 17 different days (Study name of “VAC070” in Additional file 1: Table S1). A total of 2,044%GIA values and 192 (n = 64 IgGs × 3 assays) GIA_50_ values were calculated from this trial.

An additional experiment was conducted to answer several scientific questions. Based on the available volumes, immunization groups, and activity of individual samples, individual or pooled serum samples collected from VAC057 and VAC063 trials were prepared (n = 21), and total IgGs were purified. The purified IgGs were tested at 10 or 20 mg/mL first, then 3 (n = 15 IgGs) or 4 (n = 6 IgGs) independent titration GIAs (from 20 to 0.039 mg/mL) were performed for all 21 IgGs. All GIAs were performed in singlicate wells. The assays were conducted using RBCs from 6 donors (donor Q to V) on 6 different days (Study name of “VacMix” in Additional file 1: Table S1). For the 6 IgGs samples, which were tested in 4 independent titration assays, while all %GIA values were used for analysis, the 4^th^ titration assay data (donor V) were not included for the GIA_50_ analysis to match with VAC063 and VAC070 studies where 3 titration assays were conducted. A total of 645%GIA values and 60 (n = 20 IgGs × 3 assays) GIA_50_ values were calculated from this experiment.

## Statistical analysis

The %GIA value was calculated as;

%GIA = {1- (OD_650_test – OD_650_uRBC) / (OD_650_iRBC – OD_650_uRBC)} × 100.where OD_650_test, OD_650_uRBC and OD_650_iRBC are average OD_650_ values for test antibody, uRBC and iRBC wells, respectively. The GIA_50_ value for each sample was calculated for each of 3 titration GIAs, as long as the test IgG showed > 50%GIA at the highest concentration tested in all 3 assays. The GIA_50_ value for each assay was calculated using a four-parameter logistic model with the lower asymptote parameter fixed at 0 using the L.4 function in the drc package version 3.0–1 [18] in R (version 4.2.1, The R Foundation for Statistical Computing). To evaluate a total variance and sources of variance in %GIA or in GIA_50_ measurements, linear model fits were performed using the lm function in R. For the Main GIA experiment, percent contribution of each factor to a total variance was calculated as the proportion of the sum of squared errors averaged from the two ways to order the Date and Donor variable [19]. The 95% confidence limits for the percent contributions were determined using percentile bootstrap intervals calculated with 2,000 replications by boot package (Canty A, Ripley BD (2021). *boot: Bootstrap R (S-Plus) Functions*. R package version 1.3–28.).

## Results

### Sources of EoA in %GIA

**Fig. 1:**
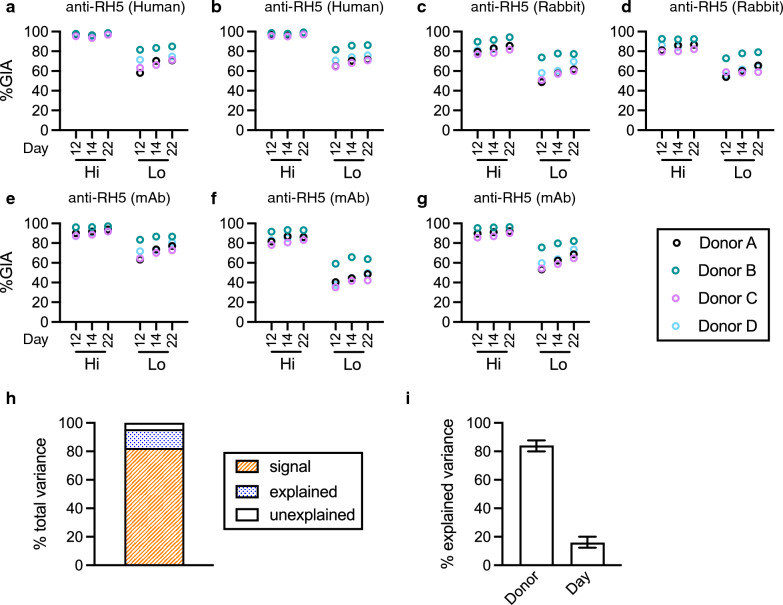
Evaluation for error of assay (EoA) in GIA with anti-RH5 antibodies. *P. falciparum* 3D7 parasites were cultured using RBCs from four different donors (A, B, C and D). Parasites, which were cultured in each donor’s RBC, were utilized to perform GIA on days 12, 14 and 22. Two human polyclonal antibodies, pAb (**a** and **b**) at 10.0 (Hi) or 2.5 (Lo) mg/mL, two rabbit pAbs at 5.0 and 1.25 mg/mL (**c**), or 10 and 2.5 mg/mL (**d**), and three human monoclonal antibodies, mAb (**e, f** and **g**) at 0.5 and 0.03 mg/mL were tested in each assay. **h** A linear model fit was performed using data shown in **a**–**g**. In the analysis, each sample at each test concentration (Sample effect), RBC donor (A, B, C or D; Donor effect) and assay day (12, 14 or 22; Day effect) were treated as independent variables. “signal” is the variance explained by the Sample effect, “explained” is a sum of variance explained by Donor and Day effects, and “unexplained” is the residual variance of the model. **i** within the “explained” variance, contributions of Donor and Day effects are shown with the 95% confidence interval (95%CI, error bars)

Four different malaria cultures were prepared where parasites in each culture were maintained using one of four different RBCs from four donors (Donor A, B, C, and D). On day 12, seven different anti-RH5 antibodies were tested at two different concentrations by GIA for each culture (i.e., a total of 4 independent GIAs were performed using the 4 different cultures on that day). The repeat assays were performed on days 14 and 22. Regardless of sample, test concentration or assay day, %GIA was generally higher with parasites cultured in RBC from donor B, while %GIA was lower with donor C’s RBC (Fig. 1 and Additional file 2: Fig. S1). Using the GIA results (a total of 168 data points), a linear model fit was conducted, where %GIA value was the dependent variable, and each sample at each test concentration (“Sample” effect hereafter), RBC batch used for culture/GIA (“Donor” effect) and assay date (“Day” effect) were independent variables. The total variance can be divided into three categories, “signal” (the variance that can be explained by Sample effect), “explained” (the variance explained by Donor and Day effects), and “unexplained” (residual of variance in the model). The “signal” variance is the one a researcher wants to measure (how much %GIA data can be explained by Sample), and the sum of “explained” and “unexplained” variances is EoA (how much %GIA data cannot be explained by Sample). The proportion of “signal” variance in the total variance was 82% (Fig. 1h), indicating the GIA could measure functional activity of test samples, although there were some errors in the %GIA readouts. The proportions of “explained” and “unexplained” variances to the total variance were 13% and 5%, respectively, and within the “explained” variance, Donor effect (84% of “explained” variance) was much larger than Day effect (Fig. 1i). The result indicates that donor-to-donor variation (tested on the same day) in %GIA was much larger than day-to-day variation (using the same donor’s RBC).

## Determination of EoA in %GIA measurement

To determine EoA in %GIA measurement more precisely, a larger data set generated from the Clinical GIA experiment was also utilized. In the Clinical GIA experiment, 180 different human anti-RH5 pAb were tested at a single concentration (first assay) or serial dilutions (titration GIAs) as described in Methods, and 5,093 individual %GIA data points were obtained (2,404 data points from VAC063, 2,044 points from VAC070, and 645 points from VaxMix, Additional file 1: Table S1). The Main GIA experiment described above was a balanced design so that each of the 14 sample/concentration pairs was assayed using the same 4 blood donors on the same 3 days. This balanced design was good for examining the sources of the variability. On the other hand, the larger Clinical GIA experiment was not balanced (i.e., on each assay day, only one donor’s RBC was used), so it was not easy to differentiate whether the EoA came from Donor, Day or both. However, because the sample size was larger (including more unique blood donors), data from the Clinical GIA experiment were also used to estimate the total EoA.

**Fig. 2:**
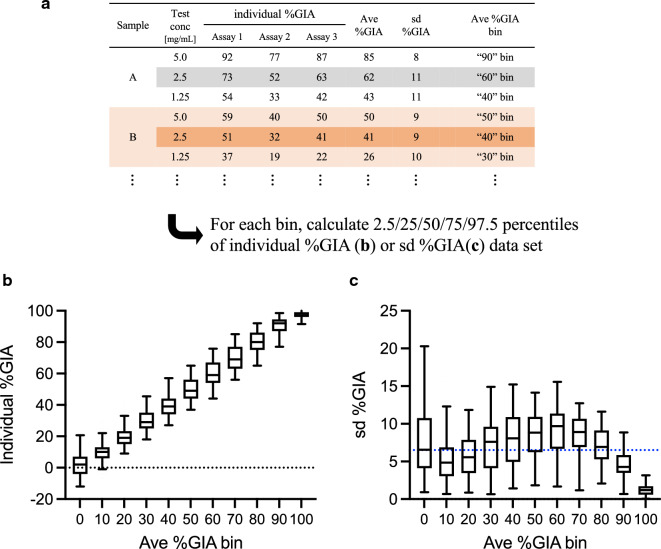
Inter-assay variability in %GIA with anti-RH5 pAb. One hundred eighty different human anti-RH5 pAbs were tested at multiple dilutions using a different batch of RBC for each assay. For each sample at each concentration tested in multiple assays, average (ave %GIA) and standard deviation (sd %GIA) values were calculated from the individual %GIA values, and then the individual %GIA and sd %GIA data points were categorized into one of 11 bins (“0” to “100” for every 10 percent point) based on the ave %GIA value. “0” ave %GIA bin contains any data from sample/concentration with ave %GIA values of < 5. “10” contains 5 $\le$ and < 15, “20” is 15 $\le$ and < 25, and so on. “100” bin contains data from ave %GIA values of 95 $\le$. **a** flow of the analysis is shown with example data sets. The box plot (25/50/75 percentiles) with 2.5/97.5 percentiles (error bars) of individual %GIA (**b**; a total of n = 5,085) or sd %GIA (**c**; a total of n = 1,638) data set for each bin are shown. The blue dotted line in **c** demonstrates an average sd of all data

Using the Clinical GIA experiment data set, average (ave) and standard deviation (sd) of %GIA from multiple assays were calculated (ave %GIA and sd %GIA) when the same sample was tested at the same concentration in multiple assays. A total of 1,638 ave %GIA values (and 1,638 sd %GIA values) was calculated from 5,085 individual %GIA data points. Then the individual %GIA and sd %GIA data for each sample at each concentration were categorized into one of 11 bins (“0” to “100” for every 10 percent point) based on the ave %GIA level (Fig. 2). Ave %GIA bin of “0” contains individual %GIA or sd %GIA data set from the sample/concentration which showed ave %GIA less than 5. Ave %GIA bin of “100” contains data set from sample/concentration which showed ave %GIA equal to or greater than 95. The EoA of %GIA measurement in an individual assay was relatively constant regardless of ave %GIA levels, except when it was closer to 0 or 100%GIA. The EoA was relatively larger for the ave %GIA bin of “0”, where test samples had no function (lower side of limit of detection of this assay). On the other hand, the EoA was relatively smaller for the ave %GIA bin of “100”, because 100%GIA is the theoretical maximum value of the assay (i.e., no parasites in the test wells). The analyses indicated that %GIA results with anti-RH5 pAb also fit reasonably well with a constant sd model, as shown in the previous study using an anti-AMA1 pAb [12], when samples were tested within the dynamic range (between lower and upper limits of quantitation) of the assay.

**Fig. 3:**
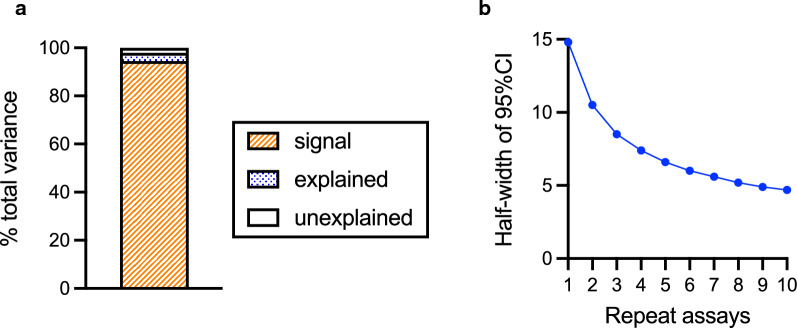
Error range in %GIA estimates. Linear model fit was performed as Fig. 1h, but all anti-RH5 antibody data (n = 5261) were used for this analysis. **b** Half-width of the 95% confidence interval (95%CI) on the %GIA of a test sample when GIA is performed in indicated number of repeat assays using different batches of RBCs on different days

Using all %GIA data (including data both from Main and Clinical GIA experiments), Box-Cox transformations were explored, and the best transformation was no transformation. Thus, non-transformed %GIA values were used as dependent variables in the following linear model fit, as performed for the Main GIA experiment. The linear model explained the observed data well (adjusted R^2^ = 0.968), and a majority of total variance (94%) was due to the Sample effect (“signal” in Fig. 3a). The sum of “explained” and “unexplained” variances was 56.9, thus the sd of assay (square-root of variance) was calculated as 7.54. The number of 7.54 was close to the average sd (6.5) in Fig. 2c. Assuming sd = 7.54, the 95% confidence intervals (95%CIs) of the true % GIA values for a test sample measured from a single or from repeat assays were calculated (Fig. 3b). When an anti-RH5 antibody is tested in a single assay, the 95% CI of the %GIA value is the estimate ± 14.8% (e.g., if a sample shows 60%GIA in a single assay, the 95%CI range is from 45.2 to 74.8%GIA). If the same sample is tested at the same concentration in three independent assays (using three different batches of RBC on three different days), the 95%CI of the %GIA value will reduce to the average estimate ± 8.5% (from $\frac{14.8\%}{\sqrt{3}}$), and the 95%CI of the %GIA value will further reduce to the average estimate ± 4.7 (from $\frac{14.8\%}{\sqrt{10}}$) if 10 independent assays are performed.

## EoA in GIA_50_ measurement

To compare functional activity among different samples, testing them at the same concentration(s) is a common method (e.g., all samples are tested at 10 mg/mL). However, the comparison might be difficult if multiple samples show ~ 100 or ~ 0%GIA at the given concentration(s). Alternatively, different samples can be compared by the concentrations that give the same level of functionality, such as GIA_50_ readout. Therefore, EoA in GIA_50_ estimates was evaluated next. In the Clinical GIA experiment, a total of 180 human pAb samples were tested at serial dilutions, but GIA_50_ values were calculated from 142 out of 180 pAb samples, because 38 pAb did not reach > 50%GIA at the highest concentration tested in at least one of repeat assays.

**Fig. 4:**
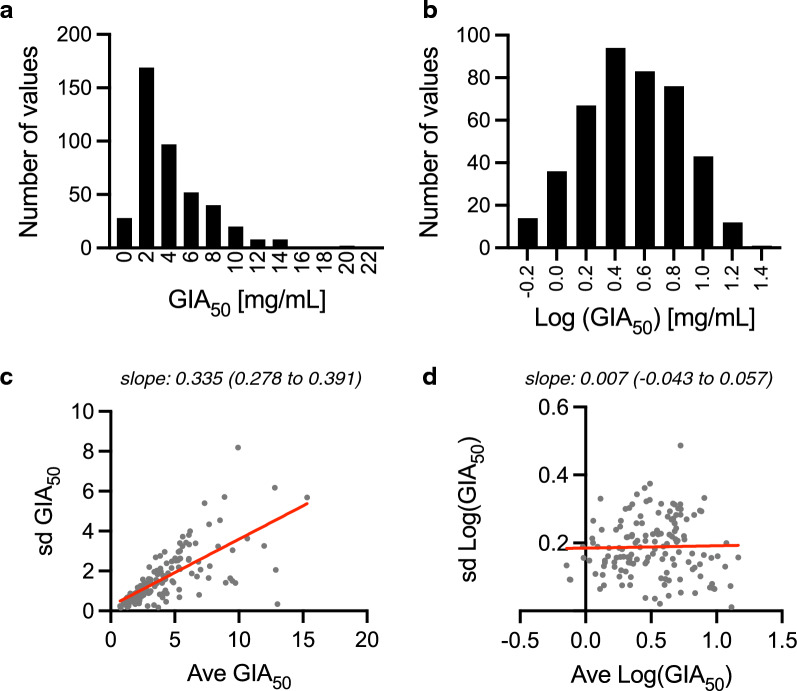
Rationale for log-transformation for GIA_50_ analyses. A total of 142 human anti-RH5 pAb were tested in 3 independent assays at serial dilutions, and GIA_50_ (total IgG concentration that gave 50%GIA) was calculated using a 4-parameter sigmoid fit (426 GIA_50_ data points). Histogram of the non-transformed (original) GIA_50_ (**a**) or log-transformed GIA_50_, Log(GIA_50_) (**b**) values are shown. **c** For each pAb sample, ave and sd of GIA_50_ from 3 independent assays were calculated. **d** The ave and sd were also determined using Log(GIA_50_) values. The red lines demonstrate linear fits and the slope (with the 95%CI) values are shown

When a histogram of (non-transformed) GIA_50_ was compared with that of log-transformed GIA_50_, the latter was closer to a normal distribution (Fig. 4a and 4b). Next, the linear relationship between average and sd in GIA_50_ measurement was investigated. The samples with higher average of GIA_50_ (ave GIA_50_) showed higher sd (sd GIA_50_) in non-transformed GIA_50_ values (Fig. 4c); the slope of best-fit line was 0.335 (95%CI; 0.278 to 0.391). On the other hand, after log-transformation, the sd (sd Log(GIA_50_)) appears independent from the average activity (ave Log(GIA_50_), Fig. 4d); the slope of best-fit line was 0.007 (95%CI; -0.043 to 0.057). Thus, the following analyses were performed using log-transformed GIA_50_ values (Log(GIA_50_)), instead of non-transformed GIA_50_ values.

**Fig. 5:**
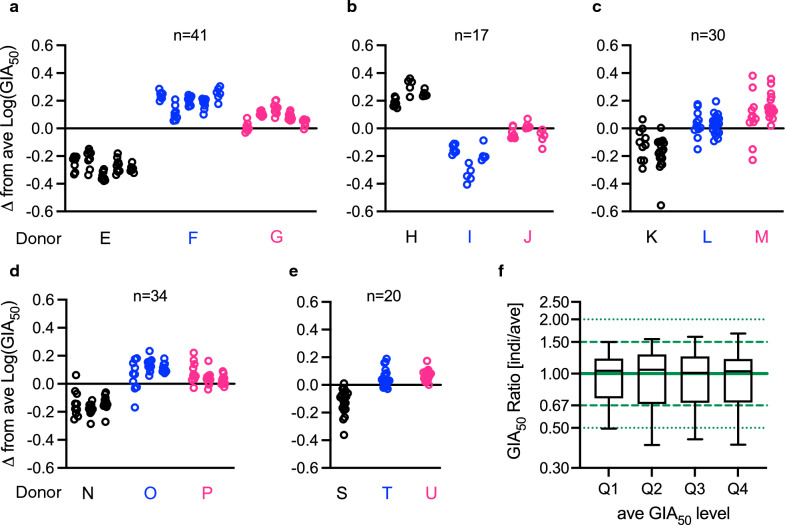
RBC donor/day effect on Log(GIA_50_) and level of EoA in GIA_50_ measurement (**a**) Indicated numbers of human anti-RH5 pAb (n = 41) were tested at serial dilutions in 3 independent assays using 3 different donors’ RBC, E, F and G. For each sample in each assay, Log(GIA_50_) was calculated, then the difference (delta) from ave Log(GIA_50_) in each assay was determined. Each dot indicates the difference for each pAb, and different columns denote data from different assay days (GIA were performed on 5 different days for each donor’s RBC). The same analysis was performed for donors’ H–J (**b**; n = 17 pAb were tested on 3 different days for each donor’s RBC), K–M (**c**; n = 30 pAb on 2 different days), N–P (**d**; n = 34 pAb on 3 different days), and S–U (**e**; n = 20 pAb on single day). **f** A ratio between (non-transformed) GIA_50_ in individual assays and the corresponding (non-transformed) ave GIA_50_ from three assays (GIA_50_ Ratio) was calculated for each sample in each assay. The ratio data were divided into 4 groups based on the ave GIA_50_ level, and each group contains data from 35 or 36 different pAb. Q1, ave GIA_50_ from 0.7 to 2.1 mg/mL; Q2, 2.2 to 3.6 mg/mL; Q3, 3.6 to 5.4 mg/mL; and Q4, 5.4 to 15.3 mg/mL. The box plot (25/50/75 percentiles) with 2.5/97.5 percentiles (error bars) for each group are shown in a log-scale figure

To visually inspect Donor and Day effects on Log(GIA_50_), a subset of samples, which were tested with donor E, F and G were selected (Fig. 5a). For each sample in each assay (i.e., for each donor/day), difference (delta) from ave Log(GIA_50_) of the three assays was calculated. As shown in Fig. 5a, a clear Donor effect was observed; regardless of assay days, Log(GIA_50_) values obtained from donor E were always lower than those from donor F or G. The same analyses were repeated for all subsets of samples for all RBCs (a subset of data for donors H, I and J shown in Fig. 5b; another subset of data for donors K, L and M shown in Fig. 5c; and so on in Fig. 5d and 5e). In general, the donor-to-donor variations were larger than the day-to-day variations within a donor (except for Fig. 5e, where day-to-day variation could not be evaluated). Due to the design of the Clinical GIA experiment, to determine contribution of Donor and Day factors in “explained” variance, as shown in Fig. 1i, was not straightforward. In other words, the % contribution of each factor in the “explained” variance changes dramatically whether Donor factor enters to a linear model before or after Day factor (data for each donor’s RBC consisted of results from multiple assay days, except for donors S, T and U, but once assay day was set, it also fixed the donor). Excluding a subset of data from donors S, T and U, a linear model fit was conducted where Log(GIA_50_) was the dependent variable, and independent variables were entered in the model in the order of Sample, Donor and Day factors. The contributions of Donor and Day effect in the “explained” variance was 94% and 6%, respectively. The result suggests that the Donor effect was likely to be larger than Day effect in the Clinical GIA experiment, as seen in the Main GIA experiment.

While using Log(GIA_50_) is reasonable for mathematical analyses, it is difficult to intuitively understand a magnitude of EoA from the numbers shown in Fig. 5a-e (e.g., what does 0.4 or -0.2 difference mean in non-transformed GIA_50_ values). Thus, the ratio (GIA_50_ ratio) between individual (non-transformed) GIA_50_ in each assay and average GIA_50_ from three independent assays was calculated for each sample (c.f., Log (A/B) = Log (A)—Log (B)). If there is no inter-assay variability in GIA, GIA_50_ ratio should be 1 (delta from ave Log(GIA_50_) should be 0). The GIA_50_ ratio data were divided into 4 groups based on ave GIA_50_ levels (Fig. 5f), and each group contained data from 35 or 36 pAb. As predicted from Fig. 4d, regardless of ave GIA_50_ level, the distribution of GIA_50_ ratio was stable. When all GIA_50_ data were combined, 70.7% of the individual GIA_50_ data points fell into between 1.5-times higher and 1/1.5-times lower (i.e., the ratio between 1.5 and 0.67) than the corresponding ave GIA_50_, and 91.5% of data points fell into between 2-times higher and ½-times lower (i.e., the ratio between 2 and 0.5).

**Fig. 6:**
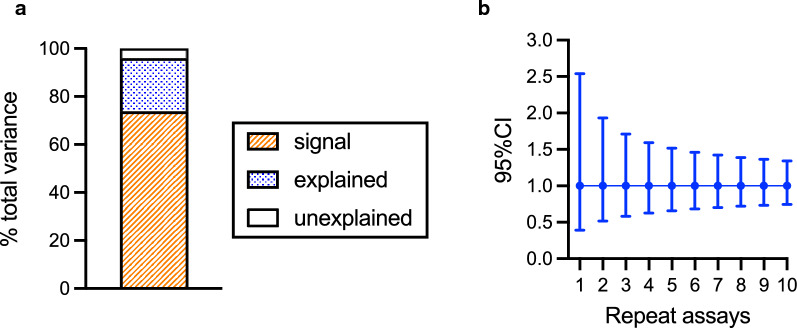
Error range in GIA_50_ estimates. **a** A linear model fit was performed as for Fig. 1h using Log(GIA_50_) as a responsible variable, instead of %GIA in Fig. 1h. **b** 95%CI of GIA_50_ for given number of repeat assays when observed geometric mean GIA_50_ is 1 mg/mL. The results are shown in a non-transformed GIA_50_ scale

To evaluate EoA in GIA_50_ measurement, a linear model fit was performed as in previous analyses, except here Log(GIA_50_) values were used as a response variable. The linear model fit reasonably well for the data set (adjusted R^2^ = 0.935). The contribution of “signal,” “explained,” and “unexplained” variance in total variance were 74%, 22% and 4%, respectively (Fig. 6a), and the sum of “explained” and “unexplained” variance was calculated as 0.0426 (i.e., sd = 0.206). Based on the value, 95%CIs of Log(GIA_50_) measurement in a single or repeat assays were calculated, then the 95%CI range was back-transformed to present 95%CI in a non-transformed GIA_50_ scale (Fig. 6b). When an anti-RH5 antibody is tested in a single assay and the GIA_50_ is measured as 1 mg/mL, the 95% CI of GIA_50_ is from 0.4 to 2.5 mg/mL. When the same sample is tested in three independent assays (i.e., using three different batches of RBC on three different days), and the geometric mean (i.e., back-transformed average of log-transformed values) of those three assays is 1 mg/mL, then the 95%CI is between 0.6 and 1.7 mg/mL, while if 10 independent assays are performed the 95% CI is between 0.7 and 1.3 mg/mL. The 95%CI (EoA) is assumed to be constant regardless of level of GIA_50_, thus 95%CI of a given GIA_50_ value in a non-transformed scale can be calculated by a simple multiplication. For example, if an observed GIA_50_ value (or geometric mean value) is 5 mg/mL, the 95%CI range is roughly between 2.0 (0.4 × 5) and 12.5 (2.5 × 5) mg/mL from a single assay, and between 3.0 (0.6 × 5) and 8.5 (1.7 × 5) mg/mL from three independent assays.

## Effect of final parasitaemia on EoA

**Fig. 7:**
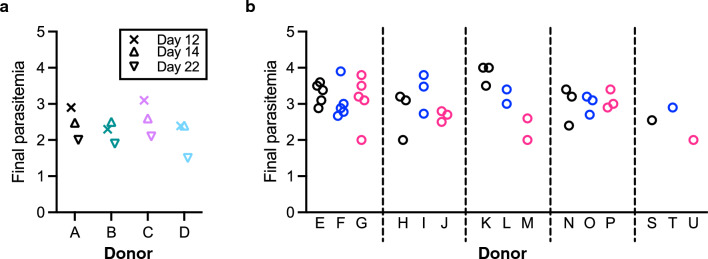
Final parasitaemia in different donors’ RBCs. Final parasitaemia in the Main GIA experiment (**a**) and the Clinical GIA experiment (**b**) are shown

To investigate a potential mechanism for the donor effect on EoA, additional analyses were performed. The working hypothesis was that parasites invaded and/or grew more efficiently in certain RBCs than in other RBCs, and the difference among different RBCs might explain higher or lower %GIA (and resulting GIA_50_) values. Since all GIAs were started at the same parasitaemia (~ 0.3%), the final parasitaemia in the negative control wells (i.e., without any test antibody) after ~ 40 h of incubation was used as an indicator for the efficiency of parasite invasion/growth. For the Main GIA experiment, while %GIA values were always higher with donor B’s RBC (Fig. 1), the final parasitaemia values in donor B’s RBC were not obviously higher or lower than those of other donors (Fig. 7a). To determine contribution of the final parasitaemia factor in “explained” variance as shown in Fig. 1i, an additional linear model fit was attempted including the final parasitaemia as one of the independent variables in addition to the Sample, Donor and Day factors analysed in Fig. 1i. However, same as discussed above for %GIA data from the Clinical GIA experiment, the analysis was not straightforward, because there was only one final parasitaemia value for a donor on a test day. When the final parasitaemia was entered into the model before the Donor and Day factors, then it contributed about 34% of the “explained” variance, while if the Donor and Day factors entered the model first, then the final parasitaemia factor amounted to only about 1% of the remaining part of “explained” variance. This means that some of the Donor and Day factor may be explained by the final parasitaemia (up to 34% of it), but also once Donor and Day are known, then final parasitaemia explains little of the remaining variability (less than 1% of the “explained” variance). Next, the final parasitaemias in the Clinical GIA experiment were also evaluated. Based on a visual inspection, there was no obvious correlation between the donor/day effect on Log(GIA_50_) (Fig. 5) and final parasitaemia (Fig. 7b). For example, when data among donors E, F and G were compared (where 5 final parasitaemia data points from 5 different assay days were available for each donor), Log(GIA_50_) values were always lower with donor E’s RBC than those with donors F and G (Fig. 5a), but the values of final parasitaemia among different assay days within donor E appeared similar to the values of the other two donors (Fig. 7b). Similarly, GIA_50_ values were higher in donor H’s RBC compared to those in donors I and J (Fig. 5b), but final parasitaemia values among three donors’ RBCs did not appear very different. When a linear model fit was conducted where Log(GIA_50_) was the dependent variable, and independent variables were entered in the model in the order of Sample, final parasitaemia, Donor and Day factors, the contributions of final parasitaemia, Donor and Day effect in the “explained” variance was 13%, 83% and 4%, respectively. Taken together, the analyses suggest that while difference of efficiency for parasite invasion and/or growth among different RBCs (indicated by the final parasitaemia) might partially contribute to the donor effect seen in this study, this factor could not explain a major part of the donor effect.

## Discussion

In this study, EoA in %GIA measurements were evaluated using a much larger data set (5,261%GIA data from 187 different samples) than the data set (one sample was tested at 6 dilutions in 4 independent assays) used for the previous study [12]. The bigger data set and an appropriate study design for the Main GIA experiment also allowed more precise evaluation of the total and each component of EoA. One of major findings of this study was that the donor-to-donor variation in GIA measurements (either %GIA or GIA_50_), which has not been recognized in the field, was much larger than the day-to-day variation. When GIA results are presented in manuscripts, the number of wells used for the assays (e.g., duplicates, triplicates) are commonly reported, and number of repeat assays are also described in many studies [13, 20–26]. However, no such studies specified whether the independent assays were performed using the same batch of RBCs or different batches of RBCs. Based on the data used in this study, sd in duplicate or triplicate wells (intra-assay variability) was relatively small (median of 1.7 with 95 percentile range of 0.2 to 5.6, n = 3,009) and constant, regardless of %GIA level (Additional file 2: Fig. S2). Therefore, well-to-well variation was not specifically evaluated in this study. If a GIA is performed with similar (or smaller) intra-assay variability as reported here, and if there is a strong donor-to-donor variation for test antibodies as observed in this study with anti-RH5 antibodies, the number of independent assays and usage of same or different batches of RBCs are much more important information than number of wells to report in future manuscripts.

Several haemoglobin genotypes and blood groups (e.g., HbSS, HbSC, HbAC, alpha-thalassaemia trait, ABO) are known to affect the invasion and/or growth rate of *P. falciparum* parasites [27, 28]. In addition, cholesterol content in RBC membrane also changes the invasion efficiency [29, 30]. Therefore, it was hypothesized that such RBC characteristics, which affected the invasion and/or growth efficiency of parasites, caused the donor effect observed in this study. Given no such RBC characteristics were assessed in this study, final parasitaemias were used as the indicator of overall invasion and/or growth efficiency of donors’ RBC. However, the linear model analyses suggested at least more than half of donor effect could not be explained by the final parasitaemia. While only limited data were available, in the Main GIA experiment, a single anti-AMA1 rabbit pAb was tested at two concentrations in each GIA plate as a positive control. Like the anti-RH5 antibody data, within the “explained” variance, the Donor effect (77% of “explained” variance) was much larger than the Day effect (23%). However, at 0.4 mg/mL concentration (as depicted as “Lo” in Additional file 2: Fig. S3), %GIA values with donor D were lower than those with the other three donors for the anti-AMA1 pAb (Additional file 2: Fig. S3), while %GIA values with donor B were higher for anti-RH5 antibodies (at higher concentration of anti-AMA1 pAb, all %GIA values were ~ 90, so there was no difference among different donors). These data suggest that the direction and magnitude of donor effect might vary even for the same donor’s RBC, depending on the targets of antibodies. If the direction of donor effect changes based on the target antigens, it becomes more difficult to assume that a major part of the donor effect comes from difference in invasion and/or growth efficiency of RBCs. Future studies are required to judge whether the strong donor effect exists for antibodies against other antigens, and to uncover the mechanism(s) responsible for this donor effect.

The 95%CI of %GIA and of GIA_50_ values measured from a single or repeat assays were also evaluated in this study. As shown in Fig. 3b and 6b, the impact of repeat assays on 95%CI range diminishes as the number becomes bigger (i.e., bigger reduction is seen from 1 to 2 assays, but almost no reduction from 9 to 10 assays). Therefore, it might not be necessary to perform > 5 repeat assays in a practical situation. However, performing at least 2 or 3 independent assays using different donors’ RBCs is ideal if researchers want to estimate true GIA activity of test samples. Having said that, while conducting 2–3 repeat assays using the same donor’s RBCs is relatively easy, performing such repeat assays using different donors’ RBCs is laborious and time consuming. Therefore, it is worthwhile to investigate whether any assay modification could simplify/shorten the assay process. For example, one option might be to culture parasites using a mixture of RBCs from three donors, then perform GIA once with the cultured parasites, instead of performing three totally independent GIAs with three donors’ RBCs separately. However, as discussed above, different RBCs in the mixture could have different invasion and growth efficiency, and %GIA readout might be dominated by a specific RBC with high efficiency. Thus, meticulous assessment is required for such modifications.

In agreement with the previous study, this study also demonstrated that %GIA data fit reasonably for a constant sd model, rather than a constant coefficient of variation (CV) model, which has been generally used for an assay to quantitatively determine concentrations of analytes (e.g., drug or metabolites in test plasmas). The constant sd in %GIA measurements and the Box-Cox transformation analysis justify usage of non-transformed %GIA values and arithmetic means to compare groups. On the other hand, for GIA_50_ analysis, this study indicates it is more appropriate to use log-transformed values, instead of non-transformed values to compare groups. In this sense, a geometric mean is better than an arithmetic mean to present GIA_50_ data from multiple assays. However, for majority of samples tested in this study, the arithmetic mean and geometric mean (and even the median) were similar (Additional file 2: Figure S4); thus practically any of the three could be used to report GIA_50_ results.

There are limitations of this study. The majority of GIA data came from assays performed with anti-RH5 antibodies (mostly human antibodies) using 3D7 clone of *P. falciparum* parasites. Therefore, it is possible that EoA determined in this study may not be applicable for antibodies from different species, antibodies against different antigens, or when GIA is performed with different strains of parasites. In the previous study, 4 independent GIA were performed against each of 3D7 and FVO parasites using one rabbit anti-AMA1 pAb (the pAb was tested at 6–7 serial dilutions in each assay) [12]. The sd was determined as 7.9 for 3D7, and 6.9 for FVO in the previous study, and these numbers largely agree with the sd determined in this study (7.54). Therefore, while additional studies are required, it is plausible to predict that the sd in %GIA measurement is around 6–8 overall, until antigen/species/strain-specific sd value is determined. The second limitation is that the constant sd model did not fit well at very low (< 10%GIA) or very high (> 80–90%GIA) levels of %GIA. However, if a test sample shows < 10%GIA, it means the sample (at the test concentration) has no functional activity. On the other hand, if another test sample shows > 80–90%GIA, for a proper comparison, the sample can be tested at lower concentration(s) so that it will show %GIA value within a dynamic range of assay (10–80%GIA range). The third limitation is that plate-to-plate and operator-to-operator variations were not assessed in this study, as none of samples were tested with the same donor’s RBC on the same assay day, but in different plates, or by different operators. Thus, a part of “explained’ variance could come from the plate-to-plate and/or operator-to-operator variations. However, the total sd is the same, regardless of contribution of each source of EoA. Therefore, this limitation should not prevent using the sd or 95%CI values determined in this study to compare different samples/groups. Lastly, the VAC070 Phase Ib trial was conducted in Tanzania, thus, %GIA and GIA_50_ data obtained from this trial could be attributed to not only anti-RH5 antibodies, but also other anti-malarial antibodies induced by natural infections. It is practically very challenging to determine how much %GIA (or GIA_50_) observed in this study came from anti-RH5 antibodies or other anti-malarial antibodies. However, in this study, only IgGs which showed >  ~ 40%GIA at the physiological concentration in the first assay were utilized (if less than ~ 40%GIA, the IgGs was tested only once, thus EoA cannot be calculated), and out of the 73 VAC70 IgGs analysed, only 3 samples collected before immunization and 1 sample collected after vaccination but from the Rabies control group reached to the ~ 40%GIA threshold [16]. Most of the IgGs came from children and infants after RH5 vaccinations, and their GIA activities strongly correlated with the anti-RH5 antibody levels measured by ELISA [16]. Taken together, it is reasonable to speculate that the majority of VAC070%GIA (and GIA_50_) data used in this study was due to the vaccine-induced anti-RH5 antibodies.

## Conclusion

GIA has been widely used to evaluate functionality of vaccine-induced antibodies, and for RH5-based vaccines, the in vitro GIA readouts have shown to be correlated with in vivo protection in non-human primates and humans. This is the first study showing there is a strong donor-to-donor variation of RBC in %GIA measurement, at least for anti-RH5 antibodies. Thus, this factor should be considered in future studies, unless a target antibody is proved to demonstrate a minimum donor-to-donor variation. The determination of EoA not only helps researchers compare GIA results from different samples/groups/studies precisely, but also guides an appropriate study design (e.g., how many samples and/or how many repeat assays are required to detect an expected difference in GIA activity with a sufficient power). Thus, this study supports future blood-stage vaccine development.

## Supplementary Information

**Additional file 1: Table S1.** Original %GIA data for each sample in each assay.

**Additional file 2: Figure S1**. Donor-to-Donor and Day-to-Day variability in %GIA. Using data shown in Fig. 1a-1g, for each sample at each concentration, averageof %GIA was calculated from 12 data points, then a differencebetween the ave %GIA and individual %GIA from each assay was calculated. Each dot represents each sample at each concentration. **Figure S2.** Intra-assay variability in %GIA. Using all data set, Ave and sd of %GIA in duplicate or triplicate wells were calculated for each sample at each concentration in each assay. Then, the sd %GIA data were grouped by the Ave %GIA levels as Fig. 2c. The box plotwith 2.5/97.5 percentilesfor each group are shown. **Figure S3.** Evaluation for EoA in %GIA with anti-AMA1 antibody. *P. falciparum* 3D7 parasites were cultured using RBCs from four different donors, and GIAs were performed on days 12, 14 and 22 as shown in Fig. 1. A rabbit anti-AMA1 antibody was tested at 2.4or 0.4mg/mL in each GIA plate as a positive control. **Figure S4.** Comparison among arithmetic mean, geometric meanand median. When the same sample was tested at the same concentration for %GIA analysis in multiple assays, ave, geomean and medium of %GIA values were calculated. The correlations among three values are shown. The similar analysis was conducted for GIA_50_ values. The blue dotted line in each panel demonstrates a y = x line.

## Data Availability

All original GIA data are presented in Additional file 1: Table S1.

## Abbreviations

GIA: Growth inhibition assay
Ab: Antibody
mAb: Monoclonal antibody
pAb: Polyclonal antibody
EoA: Error of assay
RBC: Red blood cell
%GIA: % Inhibition in GIA
GIA_50_: Antibody concentration that gave 50%GIA
sd: Standard deviation
95%CI: 95% Confidence interval
CV: Coefficient of variation
RH5: Reticulocyte-binding protein homolog 5
AMA1: Apical membrane antigen 1
MSP1: Merozoite surface protein 1
EBA-175: Erythrocyte binding antigen-175
CHMI: Controlled human malaria infection
ChAd63: Chimpanzee adenovirus serotype 63
MVA: Modified vaccinia virus Ankara
vp: Viral particles
pfu: Plaque-forming units
