# Supplementary material for: Assessment of precision in growth inhibition assay (GIA) using human anti-PfRH5 antibodies
Source: Malar J. 2023 May 19;22:159. doi: 10.1186/s12936-023-04591-6 (PMC10196285; doi:10.1186/s12936-023-04591-6)
Supplement: Supplementary file 2 — Additional file 2: Figure S1. Donor-to-Donor and Day-to-Day variability in %GIA. Using data shown in Fig. 1a-1g, for each sample at each concentration, averageof %GIA was calculated from 12 data points, then a differencebetween the ave %GIA and individual %GIA from each assay was calculated. Each dot represents each sample at each concentration. Figure S2. Intra-assay variability in %GIA. Using all data set, Ave and sd of %GIA in duplicate or triplicate wells were calculated for each sample at each concentration in each assay. Then, the sd %GIA data were grouped by the Ave %GIA levels as Fig. 2c. The box plotwith 2.5/97.5 percentilesfor each group are shown. Figure S3. Evaluation for EoA in %GIA with anti-AMA1 antibody. P. falciparum 3D7 parasites were cultured using RBCs from four different donors, and GIAs were performed on days 12, 14 and 22 as shown in Fig. 1. A rabbit anti-AMA1 antibody was tested at 2.4or 0.4mg/mL in each GIA plate as a positive control. Figure S4. Comparison among arithmetic mean, geometric meanand median. When the same sample was tested at the same concentration for %GIA analysis in multiple assays, ave, geomean and medium of %GIA values were calculated. The correlations among three values are shown. The similar analysis was conducted for GIA50 values. The blue dotted line in each panel demonstrates a y = x line. [file 12936_2023_4591_MOESM2_ESM.docx]

**Figure S1. Donor-to-Donor and Day-to-Day variability in %GIA.** Using data shown in Fig 1a-1g, for each sample at each concentration, average (ave) of %GIA was calculated from 12 data points (4 donors x 3 days), then a difference (delta) between the ave %GIA and individual %GIA from each assay was calculated (y-axis). Each dot represents each sample at each concentration (14 dots in each column).

**Figure S2. Intra-assay variability in %GIA.** Using all data set (including both Main and Clinical GIA experiments), ave and sd of %GIA in duplicate or triplicate wells were calculated for each sample at each concentration in each assay (n=3,009). Then, the sd %GIA data were grouped by the ave %GIA levels as Fig. 2c. The box plot (25/50/75 percentiles) with 2.5/97.5 percentiles (error bars) for each group are shown. The blue dotted line demonstrates a median sd of all data.

**Figure S3. Evaluation for EoA in %GIA with anti-AMA1 antibody**. *P. falciparum* 3D7 parasites were cultured using RBCs from four different donors (A, B, C and D), and GIAs were performed on days 12, 14 and 22 as shown in Fig 1. A rabbit anti-AMA1 antibody was tested at 2.4 (Hi) or 0.4 (Lo) mg/mL in each GIA plate as a positive control.

**Figure S4. Comparison among arithmetic mean (ave), geometric mean (geomean)** **and median**. When the same sample was tested at the same concentration for %GIA analysis in multiple assays, ave, geomean and medium of %GIA values were calculated (top panels). The correlations among three values are shown. The similar analysis was conducted for GIA_50_ values (bottom panels). The blue dotted line in each panel demonstrates a y = x line.
